
JOURNAL INFORMATION
==============================
NLM Title Abbreviation: J Can Health Libr Assoc
Journal ID: J Can Health Libr Assoc
NLM Journal ID: 101229936
Journal ID: JCHLA

The Journal of the Canadian Health Libraries Association

EISSN: 1708-6892
Publisher: Canadian Health Libraries Association / Association des bibliotèques de la santé du Canada

ARTICLE INFORMATION
==============================
PMCID: PMC12352439
DOI: 10.29173/jchla29894
Article ID: JCHLA-46-077
Article version: 1
Subjects: Abstracts

CHLA 2025 CONFERENCE PANELS / ABSC CONGRÈS 2025 PANELS

Electronic publication date: 2025 Aug 1
Publication date: 2025 Aug
Volume: 46
Issue: 2
First page: 77
Last page: 77
Copyright: © Fournier
Copyright year: 2025
License: This article is distributed under a Creative Commons Attribution License: https://creativecommons.org/licenses/by/4.0/
License URL: https://creativecommons.org/licenses/by/4.0/

==============================
JOURNAL INFORMATION
==============================
NLM Title Abbreviation: J Can Health Libr Assoc
Journal ID: J Can Health Libr Assoc
NLM Journal ID: 101229936
Journal ID: JCHLA

The Journal of the Canadian Health Libraries Association

EISSN: 1708-6892
Publisher: Canadian Health Libraries Association / Association des bibliotèques de la santé du Canada

ARTICLE INFORMATION
==============================
Subjects: PL = Panels

PL1. Advancing health research in Canada through open science

Foote Alyssa 1
McGrail Kimberlyn 2
Barsky Eugene 2
Hollander Zsuzsanna 3
1 World Data System,
2 University of British Columbia,
3 Genome BC

JOURNAL INFORMATION
==============================
NLM Title Abbreviation: J Can Health Libr Assoc
Journal ID: J Can Health Libr Assoc
NLM Journal ID: 101229936
Journal ID: JCHLA

The Journal of the Canadian Health Libraries Association

EISSN: 1708-6892
Publisher: Canadian Health Libraries Association / Association des bibliotèques de la santé du Canada

ARTICLE INFORMATION
==============================
Subjects: PL = Panels

PL2. Developing, validating, and using search filters to retrieve evidence related to specific populations

Parker Robin 1
Paynter Robin 2
Philippopoulos Eleni 3
Winther Connie 4
1 Dalhousie University,
2 US Agency for Healthcare Research and Quality,
3 McGill University,
4 University of Alberta

